# Arrhythmogenic right ventricular cardiomyopathy in a Japanese patient with a homozygous founder variant of *DSG2* in the East Asian population

**DOI:** 10.1038/s41439-022-00206-9

**Published:** 2022-08-08

**Authors:** Haruka Murakami, Yoko Tanimoto, Kojiro Tanimoto, Satomi Inoue, Taisuke Ishikawa, Naomasa Makita, Kazuki Yamazawa

**Affiliations:** 1 Medical Genetics Center, National Hospital Organization Tokyo Medical Center, Tokyo, Japan; 2 Department of Cardiology, National Hospital Organization Tokyo Medical Center, Tokyo, Japan; 3 Omics Research Center, National Cerebral and Cardiovascular Center, Suita, Japan

**Keywords:** Cardiomyopathies, Ventricular tachycardia, Disease genetics, Disease genetics, Genetic testing

## Abstract

Arrhythmogenic right ventricular cardiomyopathy (ARVC) is a hereditary cardiomyopathy that results in fatal arrhythmias and heart failure. Herein, we report a Japanese patient with ARVC whose parents were blood relatives. Genetic testing identified a homozygous rare variant, c.1592T > G (p.Phe531Cys), of *DSG2* that is presumed to be a founder variant among East Asians. Genetic counseling sessions with precise risk assessment and appropriate follow-up programs were provided to the patient and family members.

Arrhythmogenic right ventricular cardiomyopathy (ARVC) is a hereditary cardiomyopathy that is characterized by progressive fibrofatty replacement of the myocardium and results in malignant arrhythmias and heart failure^1^. ARVC is one of the leading causes of arrhythmic cardiac arrest in young individuals and athletes, and its prevalence is estimated to be 1 in 1000 to 5000 individuals^1,2^. The most common clinical manifestation is palpitations or effort-induced syncope in adolescents or young adults, with T-wave inversion in the right precordial leads on the electrocardiogram (ECG), ventricular arrhythmias, and right ventricular abnormalities on imaging tests. To reduce the risk of sudden death and improve the quality of life by alleviating cardiac symptoms, therapeutic options, including restricting intensive sports activity, pharmacological treatment, catheter ablation, and implantable cardioverter defibrillators (ICDs), are available^3^. From a molecular perspective, multiple genes encoding desmosomal proteins, such as *PKP2*, *JUP*, *DSG2*, *DSC2*, and *DSP*, account for ~50% of patients with ARVC in different cohorts^4,5^. Moreover, several nondesmosomal genes, such as *TMEM43*, *DES*, *PLN*, *LMNA*, *CTNNA3*, and *CDH2*, have been reported to be associated with ARVC^6^. Typically, ARVC exhibits an autosomal dominant pattern of inheritance. However, some autosomal recessive forms have been described, in which heterozygous variant carriers are healthy or only exhibit mild symptoms^7,8^. Herein, we report the first Japanese case of ARVC caused by a homozygous founder variant of the desmoglein 2 gene (*DSG2*) in an East Asian population.

The proband is a 58-year-old Japanese male with ARVC. Initially, he was diagnosed with cardiomegaly at 22 years of age, with no subsequent follow-up care conducted. At the age of 57, he visited his local doctor due to edema in his lower legs and shortness of breath. He was diagnosed with heart failure and treated with appropriate medication. Approximately 8 months later, he experienced sudden dizziness and had an episode of syncope for a couple of minutes. In addition, he experienced chest pain and discomfort and called an ambulance. On arrival at the hospital, he was diagnosed with sustained ventricular tachycardia with a heart rate of 222 beats per minute (bpm) (Fig. 1a), which was successfully treated with direct-current cardioversion. A clinical diagnosis of ARVC was reached based on the 2010 revised Task Force criteria^9^. In detail, inverted T-waves in precordial leads V_1_ through V_4_ were present on the ECG (Fig. 1b). Furthermore, magnetic resonance imaging (MRI) revealed normal left ventricular function, accompanied by mild concentric left ventricular hypertrophy. The right ventricle was dilated with a ratio of end-diastolic volume to body surface area of 117.3 ml/m^2^ (major criteria: ≥110 ml/m^2^), and the right ventricular ejection fraction was reduced to 38.5% (major criteria: ≤40%). Ectopic fat deposition at the epicardium was also noted (Fig. 1c–f). The patient underwent ICD implantation and received careful follow-up. It should be noted that the patient’s sister suddenly died of cardiomyopathy of unknown cause at the age of 27. However, this occurred over 30 years ago, and no medical records were kept. Thus, the details of her case are unknown. No other cardiac abnormality or sudden death was observed in the patient’s family, although comprehensive cardiac investigations were not conducted on the family members. Importantly, his parents were in a consanguineous marriage; that is, his paternal grandfather and maternal grandmother were blood relatives. However, the detailed information regarding their relationship was unavailable. The pedigree is shown in Fig. 2a.

**Fig. 1:**
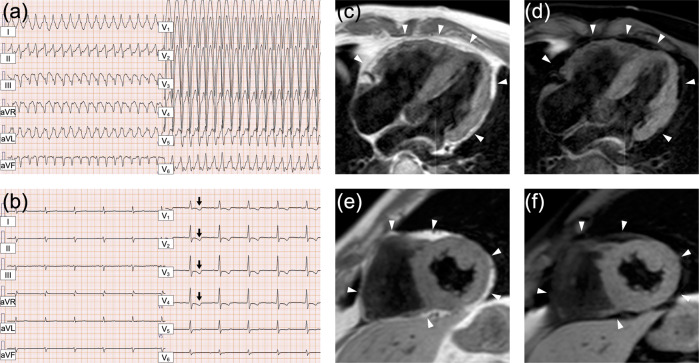
Cardiac features of the patient. **a**, **b** Electrocardiograms show ventricular tachycardia with a left bundle branch block (**a**) and negative T-waves in leads V_1_-V_4_ (arrows) (**b**). **c**–**f** T1-weighted black blood (T1WBB) magnetic resonance imaging (MRI) with (**d**, **f**) and without (**c**, **e**) spectral attenuated inversion recovery (SPAIR) to enhance the contrast of fat tissue. Four chamber views (**c**, **d**) and short-axis views (**e**, **f**) are shown. Mild concentric left ventricular hypertrophy, dilated right ventricle, and ectopic fat deposition (arrowheads) are noted.

**Fig. 2:**
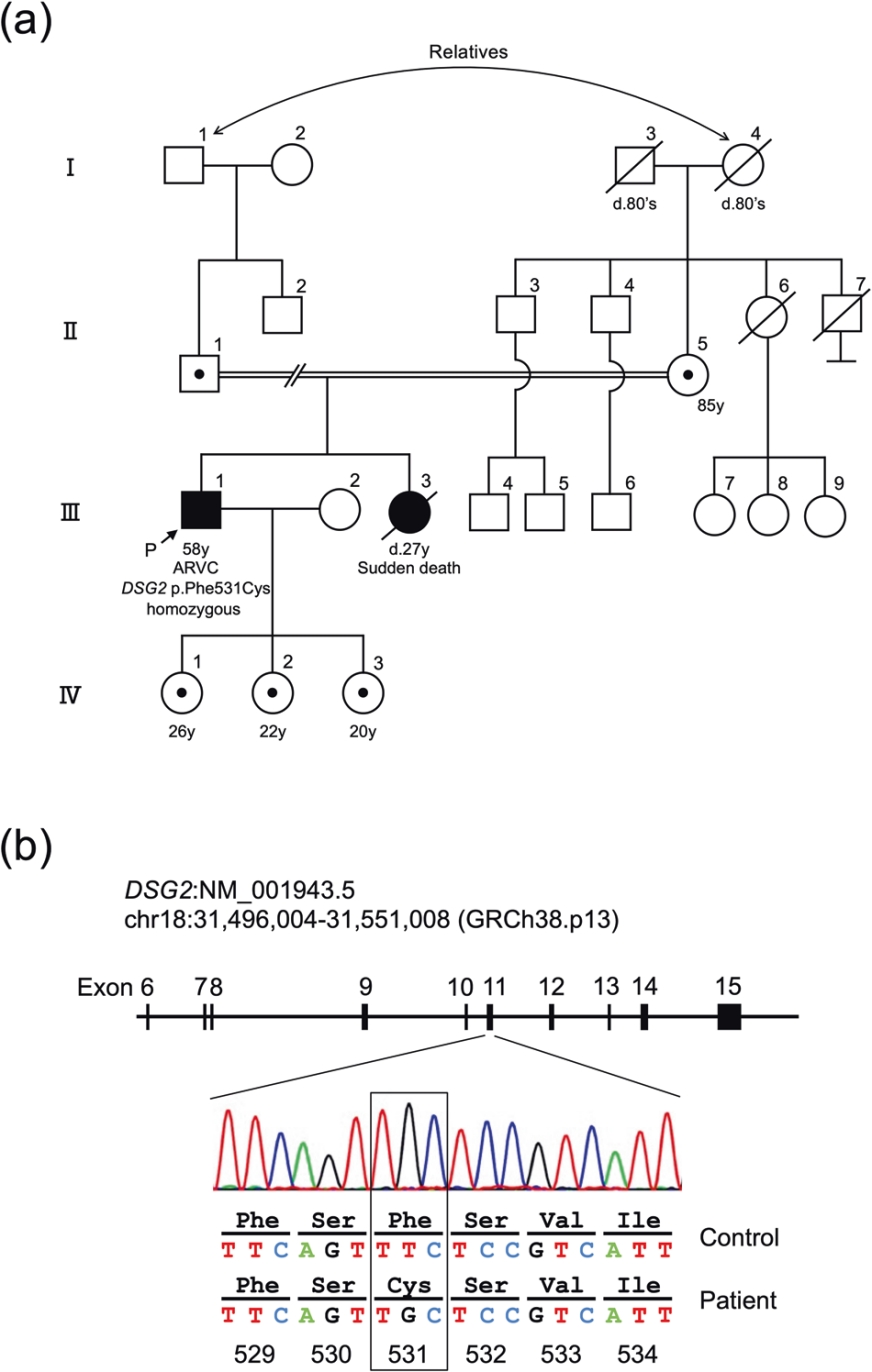
Pedigree and the variant detected in the proband. **a** The proband (III-1) was diagnosed with ARVC. His sister (III-3) died of cardiomyopathy of unknown cause at 27 years of age. No other family members had cardiac abnormalities. His parents were in a consanguineous marriage, and his paternal grandfather and maternal grandmother were blood relatives. Family history and clinical information were obtained from interviewing the proband. P: proband. Roman numerals represent generation numbers; arabic numerals indicate individual numbers. Females are represented by circles, and males are represented by squares. Obligate carriers of the *DSG2* variant are indicated by dotted squares or circles, and diagonal lines denote deceased individuals. **b** Sanger sequencing of the *DSG2* gene confirmed the presence of the homozygous c.1592T > G (p.Phe531Cys) variant in the proband.

Next, we conducted genetic testing with a multigene sequencing panel targeting 174 genes related to cardiovascular diseases (TruSight Cardio; Illumina) after providing genetic counseling and obtaining written consent. This study was approved by the institutional review board. Accordingly, a homozygous missense variant, NM_001943.5:c.1592T > G (p.Phe531Cys), in the *DSG2* gene was detected. Subsequent Sanger sequencing confirmed this finding (Fig. 2b). No other variants in genes associated with ARVC were identified.

According to ClinVar (https://www.ncbi.nlm.nih.gov/clinvar, accessed in April 2022), the interpretation of the *DSG2* p.Phe531Cys variant was “conflicting interpretations of pathogenicity”, implying that its clinical significance remains undetermined. However, this variant was evaluated as a “disease-causing mutation” in HGMD (https://portal.biobase-international.com/hgmd/pro/start.php, accessed in April 2022). For in silico analyses, multiple lines of computational evidence presented the deleterious effect of this variant (Supplementary table). The allele frequencies of *DSG2* p.Phe531Cys in the general Japanese population were 1.06 × 10^−4^ (3/28,258) according to ToMMo 14KJPN (https://jmorp.megabank.tohoku.ac.jp/202112/variants) and 8.27 × 10^−4^ (2/2,418) according to HGVD (http://www.hgvd.genome.med.kyoto-u.ac.jp). Moreover, according to gmonAD (https://gnomad.broadinstitute.org), this variant has only been reported in East Asia, with no documented evidence in other areas worldwide. The allele frequencies were 5.70 × 10^−5^ (16/280,790) in the global population and 8.19 × 10^−4^ (16/19,534) in the East Asian population (Table 1). Indeed, the *DSG2* p.Phe531Cys variant in patients with ARVC has only been reported in East Asian countries, including Japan^10^, Taiwan^11^, and China^12–15^. Among them, except for a patient from Japan who carried digenic variants of *DSG2* and *DSP*^10^, all index patients diagnosed with ARVC had the *DSG2* p.Phe531Cys variant in a homozygous manner. Collectively, these data suggest that *DSG2* p.Phe531Cys may exert a deleterious effect on the cardiac desmosome, resulting in the development of ARVC, especially when in a homozygous state. Given that 8 of 118 (6.8%) Chinese patients with ARVC carrying the homozygous variant *DSG2* p.Phe531Cys shared the same haplotype, *DSG2* p.Phe531Cys was reported as a founder variant among East Asian populations^15^. In light of the lower allele frequency of this variant in the Japanese population, homozygous variant carriers are thought to be extremely rare in Japan, except for in consanguineous families. Herein, we report the first Japanese patient with ARVC harboring the homozygous *DSG2* p.Phe531Cys variant.

**Table 1:** Allele frequencies of the *DSG2* c.1592T > G (p.Phe531Cys) variant in the general population.

Database	Population	Allele count	Allele number	Allele frequency
ToMMo 14KJPN^a^	Japanese	3	28,258	1.06 × 10^−4^
HGVD^b^	Japanese	2	2418	8.27 × 10^−4^
gnomAD^c^	East Asian	16	19,534	8.19 × 10^−4^
gnomAD^c^	Global	16	280,790	5.70 × 10^−5^
TOPMed^d^	Global	18	264,690	6.80 × 10^−5^

All data were obtained by accessing each database in April 2022.

^a^ToMMo 14KJPN Allele Frequency Panel; https://jmorp.megabank.tohoku.ac.jp/202112/variants/.

^b^Human Genetic Variation Database; http://www.hgvd.genome.med.kyoto-u.ac.jp/.

^c^Genome Aggregation Database; https://gnomad.broadinstitute.org/.

^d^Trans-Omics for Precision Medicine; https://bravo.sph.umich.edu/freeze8/hg38/.

Notably, the phenotypic variation between homozygous and heterozygous variant carriers should be addressed. To date, in the Chinese population, 19 of 19 (100%) homozygous *DSG2* p.Phe531Cys carriers were diagnosed with ARVC, and 8 of 32 (25%) heterozygous variant carriers presented with moderate ARVC symptoms at an older age. Additionally, no ARVC-related symptoms were observed in family members without the variant^15^. These percentages are assumed to be comparable to those in Japanese individuals, despite the lower allele frequency in the Japanese population. These findings provide valuable information to patients and their family members, especially for genetic counseling sessions. It was presumed that both parents of the proband had the heterozygous variant (obligate carriers), and the proband developed ARVC in an autosomal recessive manner owing to parental consanguinity. Thus, it was highly plausible that his sister, who had suddenly died of cardiomyopathy of unknown cause, also carried the homozygous variant. The patient’s daughters were 26, 22, and 20 years old and had no remarkable medical history. However, they are considered obligate carriers; thus, regular cardiac check-ups are recommended.

The limitation of this study was the lack of a detailed family survey. Indeed, family history and their clinical information were obtained from interviewing only the proband. Contacting family members or their physicians was not allowed. Thus, there still remains the possibility that some cardiac manifestations in heterozygous variant carriers may be overlooked, given that one-fourth of heterozygous carriers can develop moderate ARVC symptoms as discussed above.

In summary, we report the first Japanese case of ARVC caused by a homozygous founder variant of *DSG2* in the East Asian population that is attributed to parental consanguinity. Identifying the underlying genetic cause allowed us to provide the proband and his family members with precise genetic counseling based on an accurate assessment of the risk and appropriate cardiac intervention and/or follow-up programs.

## HGV database

The relevant data from this Data Report can be found in the Human Genome Variation Database at 10.6084/m9.figshare.hgv.3210.

## Supplementary information

Supplementary table
